# Perceptions of the Body in Cerebral Palsy: Voices of Family Caregivers

**DOI:** 10.3390/healthcare14070967

**Published:** 2026-04-07

**Authors:** Mariana Cristina Palermo Ferreira, Érica Cesário Defilipo, Lélia Cápua Nunes, Pedro Henrique Berbert de Carvalho

**Affiliations:** 1 Postgraduate Program in Physical Education (Joint Program), Federal University of Juiz de Fora and Federal University of Viçosa, Juiz de Fora 36036-900, MG, Brazil; mariana.palermo@ufjf.br; 2 Department of Physical Therapy, Federal University of Juiz de Fora, Governador Valadares Campus, Governador Valadares 35010-180, MG, Brazil; erica.defilipo@ufjf.br; 3 Department of Medicine, Federal University of Juiz de Fora, Governador Valadares Campus, Governador Valadares 35010-180, MG, Brazil; lelia.capua@ufjf.br; 4 Body Image and Eating Disorders Research Group (NICTA), Federal University of Juiz de Fora, Governador Valadares 35010-180, MG, Brazil

**Keywords:** cerebral palsy, family environment, caregivers, participation, qualitative research

## Abstract

**Highlights:**

**What are the main findings?**

- Family caregivers demonstrated ambivalent attitudes toward the participation of children and adolescents with cerebral palsy, combining encouragement with restriction.
- Support for autonomy coexisted with protective behaviors driven by fear of accidents, abuse, and social stigma.

**What are the implications of the main findings?**

- The findings highlight the need to expand family caregivers’ knowledge about cerebral palsy to mitigate limiting beliefs and promote autonomy and participation.
- These results underscore the importance of increasing societal awareness to reduce stigma surrounding the bodies of children and adolescents with disabilities and to support family-centered approaches.

**Abstract:**

**Background/Objectives**: Cerebral palsy (CP) is the most prevalent physical disability in the pediatric population, resulting in functional limitations and restrictions in participation, with higher prevalence rates in low- and middle-income countries. Caregivers of children and adolescents with CP face both physical and emotional challenges, and their perceptions of the body may act as contextual and cultural indicators shaping children’s participation, care practices, and well-being within the family environment. This study aimed to understand the perceptions, values, and cultural beliefs of family caregivers about the bodies of children and adolescents with CP. **Methods**: A qualitative study was conducted using six focus groups with 22 mothers and grandmothers of children and adolescents with CP. Participants were recruited from rehabilitation services. Discussions were audio-recorded, transcribed verbatim, and analyzed using content analysis. The analytical process involved systematic coding, categorization, and thematic interpretation to identify recurring meanings and patterns across narratives. **Results**: Three thematic categories emerged: (1) perceptions of the body within social interactions; (2) viewing the body as capable of performing activities independently when stimulated/taught; and (3) viewing the body as dependent, requiring constant supervision and support. **Conclusions**: The findings highlight the need to expand family caregivers’ knowledge about CP to promote children’s participation and mitigate beliefs related to limitations, dependence, fragility, and vulnerability.

## 1. Introduction

Cerebral palsy (CP) is an umbrella term for a spectrum of motor impairments resulting from non-progressive brain injury or malformation occurring early in life. It is a neurodevelopmental condition characterized by activity limitations resulting from disorders of movement and posture, manifesting as spasticity, dystonia, choreoathetosis, and/or ataxia. Beyond motor dysfunction, children with CP present primary and secondary impairments across multiple domains, which significantly affect their participation throughout the life course [1]. Participation comprises two key elements: attendance and involvement. Attendance refers to presence or the act of being present and encompasses the frequency and/or diversity of activities in which an individual participates, whereas involvement relates to the quality of the participation experience, including aspects such as engagement, motivation, persistence, social connection, and affect [2].

The prevalence of CP varies across countries, with rates of approximately 1.6 per 1000 live births in high-income countries, while low- and middle-income countries show higher prevalence rates, reaching up to 3.4 per 1000 live births [3]. Children and adolescents with CP exhibit heterogeneous clinical presentations, encompassing impairments, levels of functioning, and limitations in activities of daily living [1,4]. In this context, the family represents a key environmental factor for development and may function either as a facilitator or a barrier to the functioning and participation of children and adolescents with CP, through attitudes toward their child’s condition. Family relationships are shaped by reciprocal influences among family members and are influenced by social characteristics, cultural values, and physical aspects [5].

Within the family context, mothers are most often the primary caregivers of children and adolescents with CP and face significant physical and emotional challenges when dealing with complex and multifaceted realities [6,7]. Although maternal conceptions are inherently subjective, they are shaped by complex and cumulative experiences, including acceptance, coping with functional limitations, comparisons with normative developmental standards, and the broader sociocultural context. These attitudes, constructed over time, encompass perceptions, beliefs, and meanings internalized through lived experiences [7,8].

The literature presents a broad body of research on the subjective experiences of caregivers of children and adolescents with CP, primarily through qualitative studies and systematic reviews. Most studies highlight the emotional impact of caregiving [6,7,8,9,10], caregiver burden [7,9,10], challenges related to the information provided by health professionals about CP [6,7,8,9], ineffective health policies and services [6,9,10], and societal stigma [6,7,8,9,10]. Although the literature documents multiple aspects of caregivers’ subjective experiences, an important gap remains regarding how caregivers construct meanings about the bodies of children and adolescents with a disability. These perceptions, shaped by symbolic, social, and cultural meanings, may influence or restrict opportunities for participation in everyday activities among children and adolescents with CP [11,12,13].

Thus, the present study proposed the following main question: “What are the perceptions, values, and cultural beliefs of family caregivers about the bodies of children and adolescents with CP?” In this study, the concept of the body is understood as the way through which human beings experience the world, interact with others, and construct meaning through lived experience. This perspective allows us to explore how family caregivers perceive and relate to the bodies of children and adolescents with CP, capturing both the emotional and social dimensions of caregiving. Therefore, this study aimed to understand family caregivers’ perceptions, values, and cultural beliefs about the bodies of children and adolescents with CP.

## 2. Materials and Methods

### 2.1. Study Design

This study employed a descriptive, exploratory study design with a qualitative approach, utilizing focus groups as the primary data collection technique [14]. This type of research enables reflection on and understanding of the phenomena under investigation through group discussions based on participants’ lived experiences, fostering the generation of insights [14].

### 2.2. Participants

The study included family caregivers of children and adolescents diagnosed with cerebral palsy (CP). Six focus groups were conducted between April and July 2024, involving 20 biological mothers and two grandmothers. Three focus groups comprised nine mothers and two grandmothers of children and adolescents classified at levels I to III of the Gross Motor Function Classification System (GMFCS) [15], while the remaining three groups included 11 mothers of children and adolescents classified at GMFCS levels IV and V. The number of focus groups was determined based on the criterion of theoretical data saturation, defined as the point at which data collection no longer yielded new or relevant information, indicating that the phenomenon under study had been sufficiently explored [14].

### 2.3. Instruments

A semi-structured guide with topic prompts was used during the focus groups to encourage and facilitate discussion about mothers’ and grandmothers’ perceptions, values, and beliefs regarding the bodies of children and adolescents with CP, and the influence of these perceptions on participation. The semi-structured guide was developed by the authors based on a review of the literature on the topic and was subsequently submitted for content validation by four experts in the field of child development [14]. The semi-structured guide was revised based on the experts’ suggestions and subsequently used in the focus groups (please see Supplementary Materials).

At the end of each focus group, a questionnaire was administered to collect information on participant characteristics, family context, and characteristics of the children and adolescents. The questionnaire comprised two sections: (1) a sociodemographic section, including the *Critério de Classificação Econômica Brasil* developed by the *Associação Brasileira de Empresas e Pesquisa* [16], to determine the family’s socioeconomic status; and (2) a section addressing CP classification systems, including the GMFCS [15] and the Manual Ability Classification System (MACS) [17] or the Mini-MACS [18], according to the child’s age. According to the literature, these classification systems demonstrate adequate reliability when reported by families of children and adolescents with CP [18,19,20].

### 2.4. Procedures

#### 2.4.1. Data Collection

Initially, the lead researcher contacted by telephone the family caregivers of children and adolescents with CP receiving care at the Physical Therapy Teaching Clinic of the *Universidade Federal de Juiz de Fora*, as well as at private clinics and public services providing care in the municipality of *Governador Valadares* (*Minas Gerais*, Brazil). Participant selection was based on a convenience sample combined with snowball sampling techniques [14].

During the initial telephone contact, the study was explained, and family caregivers were invited to participate. At this stage, only one mother declined participation, citing insecurity and shyness regarding group discussions. For those who agreed to participate, availability regarding date and time was assessed to schedule the focus group sessions. Focus group scheduling was organized according to the five functional ability levels of children and adolescents with CP, as classified by the GMFCS [15]. This division was adopted based on the assumption that families’ perceptions of the bodies of children and adolescents under their care could vary according to functional ability levels.

The focus groups were conducted in a private, air-conditioned room, designed to be welcoming and secure, with appropriate equipment and furniture, at the Physical Therapy Teaching Clinic of the *Universidade Federal de Juiz de Fora*. Two Sony^®^ (Manaus, Brazil) digital voice recorders (ICD-PX240) were placed on benches in the center of the discussion circle, and a Samsung^®^ (Manaus, Brazil) mobile phone was used for video recording. The camera was mounted on a tripod and positioned in the corner of the room, allowing for frontal capture of most participants.

Each focus group was facilitated by two moderators and one observer. The moderators were seated in a circle with the participants: the lead moderator was a physical therapist with a master’s degree and professional experience in child development, and the co-moderator was a nutritionist with a doctoral degree and academic experience in conducting focus groups. The observer, a physical therapist with a doctoral degree and academic experience in child development, was seated opposite the camera position, with the purpose of observing non-verbal communication and taking additional field notes. All three members of the research team were mothers of typically developing children and identified as cisgender women.

Upon arrival at the focus group location, participants were welcomed by the lead moderator, introduced to the research team, and provided with the informed consent form for reading. After signing the informed consent form, the lead moderator introduced herself and explained the objectives of the group, emphasizing the safe environment and assuring the anonymity and confidentiality of the discussions. Subsequently, the co-moderator and the observer introduced themselves. Participants then introduced themselves and shared a characteristic of children or adolescents under their care, highlighting a favourite play activity. Following this initial moment of rapport-building and familiarization, reflections, discussions, and shared experiences began, either spontaneously among participants or prompted by guiding questions from the semi-structured guide, facilitated by the lead moderator.

Before concluding each session, the lead moderator invited participants to share additional comments regarding the discussions or topics that had not been addressed and asked them about their perceptions of participating in the group. The session was then concluded with expressions of gratitude, and audio and video recordings were stopped. After the focus groups ended, participants completed the questionnaire, and any questions were clarified by the research team. The focus groups lasted between 80 and 120 min, with a mean duration of 100 min.

#### 2.4.2. Data Analysis

The focus group recordings were transcribed verbatim in three stages. The first stage involved initial transcription based on the audio recordings. The second stage consisted of reviewing the initial transcription using the video recordings, adding further detail related to participants’ non-verbal communication. The third and final stage involved revising the transcripts using both audio and video recordings to ensure content accuracy. Field notes recorded by the observer during the focus groups were incorporated into the transcripts to enrich the interpretation of non-verbal communication and contextual details.

Data were analyzed using Content Analysis [21], conducted in three stages: (1) pre-analysis, involving organization of the material, initial reading, and systematization of preliminary ideas; (2) material exploration, including the definition of thematic categories and recording units; and (3) treatment of results, inference, and interpretation, synthesizing the information generated by the analysis and enabling inferential interpretation. Descriptive analyses of participant, child, and adolescent characteristics were performed using Jamovi software, version 2.6 (The Jamovi Project) (available at https://www.jamovi.org).

#### 2.4.3. Ethical Considerations

The study was approved by the Research Ethics Committee of the *Universidade Federal de Juiz de Fora* (protocol code: 74847623.9.0000.5147; approval number: 6.586.119). To ensure participant anonymity, identification codes were used. These codes comprised the participant number (e.g., P1, P2, P3), followed by their relationship to the child or adolescent (mother or grandmother), the child’s sex and age, and the GMFCS level.

## 3. Results and Discussion

The main characteristics of the participants, children and adolescents are presented in Table 1. In addition, 59.1% of the participants self-identified as brown/mixed-race, 22.7% as Black, and 18.2% as White. Regarding the families’ socioeconomic status, 4.5% were classified as high income, 41% as medium income, 50% as low income, and 4.5% as very low income, according to ABEP [16]. Most participants were married or in a stable union (54.5%), 27.3% were single, and 18.2% were divorced.

Regarding children and adolescents, the mean age was 8.32 years (SD = 3.15); 17 were male (77.3%), 20 attended daycare or school (90.9%), and four of them did not have an aide/monitor (20%). Concerning the type of muscle tone, a large proportion of participants were unable to specify (59.1%), eight reported spastic tone (36.4%), and one had mixed tone (4.5%). Regarding topographical distribution, 59.1% were bilateral, 22.7% unilateral, and four participants could not identify their distribution (18.2%). The vast majority used one or more assistive technologies (81.8%), such as a wheelchair, shower chair, walker, standing frame, cervical collar, and orthosis. Concerning manual function, classified by MACS or Mini-MACS, 4.5% were level I, 27.3% level II, 31.8% level III, 18.2% level IV, and 18.2% level V.

These findings are consistent with recently published studies from the multicenter cohort study conducted in Brazil, PartiCipa Brazil. These studies also reported a higher prevalence of male children and adolescents with CP and a predominantly bilateral topographical distribution [4,22]. It is noteworthy that in the study conducted in the state of Minas Gerais, most family caregivers of children and adolescents with CP had at least completed high school, and a large proportion were unable to specify their child’s muscle tone, with spastic tone being the most frequently reported [4]. Furthermore, there was a higher prevalence of children and adolescents classified at levels II and V of the GMFCS [4]. All of these characteristics were also observed in the population of the present study.

In the content analysis, three thematic categories emerged regarding perceptions, values, and cultural beliefs about the bodies of children and adolescents with CP. These were: (1) perception of the body in social interactions; (2) viewing the body as capable of performing activities independently when stimulated/taught; and (3) viewing the body as dependent, requiring constant supervision and support. The thematic categories and corresponding codes derived from the analysis are presented in Table 2 and discussed below.

### 3.1. Perception of the Body in Social Interactions

The first category encompasses reports that highlight family caregivers’ discomfort and distress regarding the way others in their daily routine—whether strangers, acquaintances, or family members—express themselves about the child’s or adolescent’s body, often marked by prejudice, rejection, and estrangement. In such situations, family caregivers reported feelings of suffering, anger, and emotional distress, as illustrated in the following statements:

*“And when he experiences some kind of prejudice, which is almost daily… we notice it, but he doesn’t. So, he doesn’t suffer. Who suffers is us (slight laughter).”*

(P1, mother, boy, 9 years, GMFCS II)

*“Sometimes… even if you arrive somewhere and are well received, you have different eyes on you, right? I HATE (anger) that people would come near me and say, ‘Oh, poor thing!’”*

(P10, mother, boy, 7 years, GMFCS V)

*“I faced many challenges and rejection from my own family (crying), having to say that my daughter is crippled, that my daughter was nothing, no one, so that is why I get very emotional when I talk about having suffered so much.”*

(P8, mother, girl, 12 years, GMFCS IV)

The stigma imposed by society regarding physical appearance related to the bodies of children and adolescents with CP, seen as social disharmony, leads to rejection and social discrimination [7,9]. Lack of knowledge about the disability and societal constructions regarding its cause contributes to harmful beliefs. The use of inappropriate terms such as “disabled,” “incapable,” or “crippled” directly affects families [7]. Negative attitudes—whether through comments, looks, or even laughter—make mothers feel powerless in protecting their children and hurt, contributing to social isolation [7,8,11].

Consequently, caregivers increasingly face difficulties in maintaining their own interests and social connections, limiting the family’s participation in spaces outside the home, even with close relatives [7]. This lack of societal understanding and support is a major source of maternal psychological and physical stress. Experiencing a sense of lost freedom, mothers feel alone and solely responsible for meeting all the demands of their children, reporting negative emotions such as sadness, disappointment, fear, and anger [8,10].

Some family caregivers also reported situations of prejudice, rejection, and estrangement regarding the bodies of children or adolescents under their care. However, their narratives revealed that they do not internalize the opinions or concepts imposed by others, resisting interference in how they express themselves and act, and defending a new social understanding of bodies of children and adolescents under their care. In this regard, the following statements are noteworthy:

*“When she was born, and because of her difficulties, I started showing her that she cannot hold prejudice against anyone; if someone has prejudice against her, she doesn’t need to curse or fight. She can just let it go.”*

(P4, grandmother, girl, 7 years, GMFCS I)

*“I’ve heard people telling me I should have another child, to have another experience. The experience I have is this one, as a mother of a child with atypical development. People think I need another child to feel like a mother. My experience is this.”*

(P18, mother, boy, 8 years, GMFCS V)

Bistaraki et al. [8] state that the actions adopted by caregivers of children with CP in moments of discrimination and prejudice depend on how they manage their own emotions and how they perceive and accept their children’s abilities, capacities, and vulnerabilities. As in the present study, indifference, protection, and self- and child-support are some of the strategies employed to cope with stigma.

### 3.2. Viewing the Body as Capable of Performing Activities Independently When Stimulated/Taught

The second category was systematized by observing multiple reports from family caregivers regarding their desire for their children and grandchildren to acquire greater independence and autonomy, both at home and in external environments. Participants described in detail how they provide daily stimulation through verbal prompts, imitation, and training in activities of daily living (ADLs). The most frequently mentioned activities included meal preparation, eating, washing dishes, bathing, making the bed, and dressing/undressing.

These reports demonstrate the belief that the body of a child with CP can perform activities independently after stimulation and learning, a perception different from that reported by family caregivers regarding their children’s bodies in social interactions. A mother of a 15-year-old boy (GMFCS II) reported:

*“[…] at lunchtime, I tell him to put the juice on the table. He gets the cups, places them on the table, these kinds of things… I am already giving him this instruction, and then he does it (laughs).”*

Another described teaching her son a self-care activity:

*“[…] I am starting to teach him to rub his body by himself, and then he rubs [imitates the movement with her right hand], because he knows how to rub his stomach (laughs).”*

(P16, mother, boy, 3 years, GMFCS I)

Other reports explicitly highlight the pursuit of their children’s autonomy:

*“What I could do and what I can do for his independence, this is what I show him: ‘Independence, my son, you need to have your independence.’ For example, dishes—he washes the dishes at home. He climbs onto the stool. I taught him… the plates, the cutlery, the cups [demonstrates movements with her hands]. First, you wash like this… I kept teaching him.”*

(P2, mother, boy, 9 years, GMFCS II)

*“She eats by herself. When she wants more, and I see the pans are cold, I say, ‘No, go put it yourself.’ Then I tell her, ‘Do you want to eat more? Then go put it for yourself.’ Then she goes, holding onto the wall, with her plate, and places it herself [demonstrates the movement with her hands].”*

(P13, mother, girl, 11 years, GMFCS III)

Novak et al. [23], in a systematic review on best interventions in CP, present substantial data from clinical trials demonstrating the effectiveness of task-specific and goal-directed training interventions to improve function, task performance, and motor learning, such as action observation training, task-specific training, and home-based goal-directed programs. Therefore, it is extremely important for families to receive proper guidance and to stimulate their children to perform ADLs with greater independence and autonomy in real-life contexts [24]. Considering children’s and adolescents’ preferences and interests when selecting activities facilitates greater participation through motivation and engagement [12].

Some family caregivers emphasized the importance of environmental and equipment adaptations to enable greater independence in ADLs, such as toileting and feeding:

*“We made an improvised potty, which I always leave in the bathroom. Then he crawls there, takes off his underwear, uses the potty, flushes, dries himself, puts the underwear back on, and leaves, all crawling [demonstrates the movements with hands]. We removed all obstacles to make it easier for him.”*

(P11, mother, boy, 13 years, GMFCS IV)

*“If I place him in a corner on the floor, put the small table in front, then he puts his arm, and if I put something there for him to eat, he eats alone, drinks from his cup with a straw, you know?”*

(P17, mother, boy, 7 years, GMFCS IV)

It is noteworthy that these family caregivers had children with lower gross motor function (level IV) and yet routinely ensured adaptations to allow for greater autonomy and participation during ADLs at home. Environmental, equipment, or activity-rule adaptations facilitate greater participation of the child in real-life activities [12,24].

Reports also highlighted family caregivers encouraging greater autonomy during transfers and mobility at home and in external environments. One report illustrates that even when mothers are expected to assist, autonomy can still be stimulated:

*“[…] It’s like when he fell, for example, people would say, ‘Mom, aren’t you going to lift your child?’ ‘No, he is learning to get up, he will get up by himself.’”*

(P2, mother, boy, 9 years, GMFCS II)

*“He goes to the bathroom, knows how to get there. Sometimes he drags himself, but I say, ‘Not like that! Let’s go!’ Then he starts to lean on the wall. I step aside, give him space to pass, and then he goes. You see?”*

(P22, grandmother, boy, 4 years, GMFCS I)

Additionally, this category includes reports of family caregivers encouraging participation in activities in external environments, recognizing the importance of socialization with peers in places such as squares, parks, parties, pizzerias, shopping malls, and clubs. For example:

*“The last time I took him to the mall to play, he managed on his own. Of course, I pay attention, right? But I let him go, climb the ramp, play on the equipment, I just observe, but he handles it himself.”*

(P22, grandmother, boy, 4 years, GMFCS I)

One mother emphasized her child’s happiness when interacting with other children:

*“And in places, too, when he wants to join the other children, I let him go among them. I take him out of the chair and place him on the floor. I let him go because I prefer that he plays and sometimes gets a little hurt here or there, but he doesn’t mind, seems not to feel pain. Because the happiness of being there, interacting, and playing is greater than any pain he might feel.”*

(P11, mother, boy, 13 years, GMFCS IV)

These statements highlight family caregivers’ perceptions of the importance of play, enabling feelings of autonomy and belonging in their children during various activities with peers, despite physical risks. Studies show that socialization and positive peer relationships are important factors for participation. Sports, games, and art- or culture-related activities are described as leisure options that provide joy for children and adolescents with CP [12,25].

### 3.3. Viewing the Body as Dependent, Requiring Constant Supervision and Support

The third and final category was characterized by numerous family caregivers reports regarding the perception of a dependent body, which presents limitations in almost all activities of daily living, requiring constant care, supervision, and support. This perception of dependency was internalized by participants, resulting in the creation of barriers to performing tasks independently, even for partial completion of an activity. Consequently, family caregivers increasingly adopt overprotective behaviors in their daily routines, generating restrictions on their children’s participation:

*“He cannot kick a ball, it is very difficult for him to catch it, right? So I have to be there doing it for him. He depends a lot on me.”*

(P10, mother, boy, 7 years, GMFCS V)

Other examples include the following:

*“Since he is unable to go or reach, we have to do it for him.”*

(P12, mother, boy, 3 years, GMFCS V)

*“He cannot bathe alone, cannot brush his teeth. So, I have to do everything for him.”*

(P18, mother, boy, 8 years, GMFCS V)

It is noteworthy that most reports about the perception of a dependent body came from family caregivers of children with lower functional abilities (GMFCS IV and V). However, this perception was also observed in some mothers with children who had higher gross motor skills, as illustrated in the following statement:

*“He depends solely and exclusively on me.”*

(P1, mother, boy, 9 years, GMFCS II)

This constant need for care can generate a sense of interdependence between mother and child. The following statement illustrates maternal overprotectiveness, with the mother perceiving herself as an extension of her child’s body, fully assuming her child’s bodily functions and mediating the child’s relationship with the world:

*“If we could, we would put her back inside my belly. To protect her, like this, I won’t let her fly, so that nothing happens to her!”*

(P6, mother, girl, 6 years, GMFCS V)

Qualitative studies exploring parents’ perspectives on the activities and participation of children with CP similarly describe this perception of dependency and overprotection. Mei et al. [26] indicated that parents of children with CP identified aspects of their interaction with their children as barriers, anticipating their needs rather than allowing children to express them, thereby limiting activity and restricting participation. Schiariti et al. [27] reported that some caregivers admitted restricting their children’s activities to maintain safety, while recognizing that the children might be capable of doing more than permitted.

Therefore, parental empowerment, as a practice of family-centered care, is crucial. Informed and confident parents are more engaged in daily care, involve their children in everyday activities, provide greater learning opportunities, and encourage independence. Kalleson et al. [24] found a positive relationship between parental empowerment and the frequency of participation in daily family activities for children with CP during early childhood. Families who perceive themselves as key agents in their children’s development create an environment conducive to participation in daily activities.

Additionally, some family caregivers reported concerns about an unstable body, with increased susceptibility to falls and accidents, resulting in protective behaviors or avoidance of certain situations, such as fear of their child sleeping at a grandparent’s house due to stairs. One report refers to school pickup:

*“I preferred to pick her up… at two o’clock, right? Because at two-thirty, I think they release the kids. Since it’s on a hill, right? To have time to go down. So I go down with her calmly, slowly. Without the kids risking bumping into her and her falling! The kids go down running.”*

(P13, mother, girl, 11 years, GMFCS III)

A recurring feeling of fear in caregiving was observed. Although grounded in real risks, this fear—also experienced in the development of children without disabilities—reflects the persistent belief in the fragility and vulnerability of a child with disabilities. Marques et al. [11] similarly reported caregivers associating fear of falls and accidents with restriction of activities, such as cycling or playing ball.

Focus group discussions also addressed caregivers’ concerns about the risk of sexual abuse when children are not under supervision, adding to the emotional burden of care. This perception, a potential barrier to participation in external environments such as school, is illustrated in the following statement:

*“And at school, I also didn’t want to send him because I was really afraid. Really afraid of mistreatment, of not watching him. Even regarding touching, I taught him to tell me if anyone touched him there. Once he learned, I sent him to school. I feared that someone could harm him, and he wouldn’t know how to defend himself. In my mind, I am his shield! So, if the shield isn’t there, who will protect him?”*

(P11, mother, boy, 13 years, GMFCS IV)

It is important to note that reports of this type of violence came from family caregivers of children who do not communicate verbally. A recent systematic review with meta-analysis showed that children with disabilities are twice as likely to experience multiple forms of violence, including sexual abuse, compared to their peers without disabilities [28]. Thus, the difficulty in trusting people and institutions highlights the fragility of child protection networks, making maternal protection a key mechanism against sexual violence.

Finally, it is important to consider the gendered nature of family caregiving. All caregivers included in this study were women, reflecting broader societal patterns in which women disproportionately perform unpaid caregiving work. From a feminist political economy perspective, this gendered care not only shapes caregivers’ daily experiences but also has broader implications: it can constrain women’s participation in the paid economy, contribute to emotional and physical burden, and impact their overall wellbeing, as well as that of the individuals under their care [29,30]. These contextual factors should be taken into account when interpreting our findings.

This study has limitations that should be considered. The sample consisted of mothers and grandmothers of children and adolescents with CP attending rehabilitation services in one municipality. Mothers and grandmothers from other regions, or whose children do not attend rehabilitation services, may have different perceptions and beliefs, which should be considered in other contexts. In addition, the focus groups mostly included mothers and grandmothers of boys; a more balanced sample of male and female participants would be an improvement for future research.

Despite these limitations, this study has several notable strengths. The use of focus groups enabled the collection of detailed perceptions from family caregivers, including mothers and grandmothers of children and adolescents with varying levels of motor function. The in-depth thematic analysis identified categories reflecting nuances of family caregiving, the possible autonomy of children, and perceived dependency, contributing to the understanding of caregiver burden and strategies. Furthermore, the findings provide practical insights for health professionals, public policies, and family support programs, underscoring the social and academic relevance of the study.

## 4. Conclusions

The study contributes to the knowledge and understanding of family caregivers’ perceptions and beliefs regarding the bodies of children and adolescents with CP. In some statements, it was possible to identify moments in which family caregivers encouraged behaviors that fostered autonomy and participation in their children and grandchildren, while in others, restrictions to such participation were observed, motivated by fear of accidents and abuse. From this perspective, the findings highlight the need to expand caregivers’ knowledge about CP to mitigate beliefs related to limitations, dependency, fragility, and vulnerability, as well as to raise societal awareness to reduce stigma surrounding the bodies of people with disabilities. Encouraging positive attitudes, empathy, and parental empowerment are strategies that can positively influence caregivers’ physical and mental health. The adoption of a family-centered care approach by health professionals is essential for promoting the holistic development and participation of children and adolescents with CP, ensuring their rights and strengthening protection networks for this population.

## Figures and Tables

**Table 1:** Characteristics of the participants, children, and adolescents.

Participant	Age	Education	Family Caregivers Relationship	Child/Adolescent Age	GMFCS *
P1	38	High school	Mother	9	II
P2	43	Middle school	Mother	9	II
P3	31	Higher education	Mother	9	II
P4	50	Middle school	Grandmother	7	I
P5	50	Elementary school	Mother	13	IV
P6	30	High school	Mother	6	V
P7	43	Elementary school	Mother	9	V
P8	35	High school	Mother	12	IV
P9	42	Higher education	Mother	7	V
P10	31	High school	Mother	7	V
P11	39	Elementary school	Mother	13	IV
P12	29	High school	Mother	3	V
P13	36	Elementary school	Mother	11	III
P14	41	Middle school	Mother	15	II
P15	39	Elementary school	Mother	10	II
P16	38	Higher education	Mother	3	I
P17	36	High school	Mother	7	IV
P18	29	Middle school	Mother	8	V
P19	47	High school	Mother	7	V
P20	39	Higher education	Mother	6	III
P21	48	Higher education	Mother	8	III
P22	54	Higher education	Grandmother	4	I

* GMFCS = Gross Motor Function Classification System; distribution: level I (13.65%), level II (22.7%), level III (13.65%), level IV (18.2%), and level V (31.8%).

**Table 2:** Codes derived from content analysis of family caregivers.

Thematic Category	Code	Description
Perception of the body in social interactions	Social stigma and prejudice	Caregivers report negative attitudes, prejudice, or discriminatory behaviors from strangers, acquaintances, or family members toward the child’s body.
	Emotional distress of caregivers	Feelings of sadness, anger, fear, or frustration experienced by caregivers due to societal reactions to their child’s body.
	Family rejection	Experiences of negative judgment, exclusion, or lack of support from extended family members.
	Resistance to societal judgments	Caregivers actively resist internalizing others’ opinions and defend their own understanding of their child’s body and abilities.
Viewing the body as capable of performing activities independently when stimulated/taught	Encouragement of independence	Caregivers promote the child’s autonomy through verbal guidance, demonstration, and daily encouragement.
	Training in activities of daily living (ADLs)	Caregivers teach daily self-care and household tasks, such as eating, bathing, dressing, making the bed, and meal preparation.
	Environmental adaptations	Modifications to the environment or use of assistive equipment to facilitate independent participation in daily activities.
	Participation in mobility and transfers	Encouraging the child to move and transfer independently at home and in external environments.
	Social and recreational participation	Encouraging interaction with peers and participation in outside activities, such as play, parks, parties, and shopping, promoting socialization and sense of belonging.
Viewing the body as dependent, requiring constant supervision and support	Perceived dependency	Caregivers perceive the child as unable to perform most activities independently, even partially.
	Overprotective behaviors	Caregivers adopt excessive protective actions, restricting the child’s autonomy and participation in daily activities.
	Fear of accidents or falls	Caregivers experience fear related to falls, injuries, or other risks, leading to restricted participation in daily or social activities.
	Concerns about abuse	Caregivers are vigilant about potential sexual or physical abuse, especially when children are not under supervision, limiting external participation.

## Data Availability

The data that support the findings of this study are not openly available due to reasons of sensitivity and are available from the corresponding author upon reasonable request.

## Supplementary Materials

The following supporting information can be downloaded at https://www.mdpi.com/article/10.3390/healthcare14070967/s1, Chart S1: Interview guide.

## Abbreviations

The following abbreviations are used in this manuscript:

CP	Cerebral palsy
GMFCS	Gross Motor Function Classification System
ABEP	Associação Brasileira de Empresas e Pesquisa
MACS	Manual Ability Classification System
ADLs	Activities of daily living

## References

[1] Dan B., Rosenbaum P., Carr L., Gough M., Coughlan J., Nweke N. (2025). Proposed updated description of cerebral palsy. Dev. Med. Child. Neurol..

[2] Imms C., Adair B., Keen D., Ullenhag A., Rosenbaum P., Granlund M. (2016). ‘Participation’: A systematic review of language, definitions, and constructs used in intervention research with children with disabilities. Dev. Med. Child. Neurol..

[3] McIntyre S., Goldsmith S., Webb A., Ehlinger V., Hollung S.J., McConnell K., Arnaud C., Smithers-Sheedy H., Oskoui M., Khandaker G. (2022). Global prevalence of cerebral palsy: A systematic analysis. Dev. Med. Child. Neurol..

[4] Chagas P.S.C., Lemos A.G., Ayupe K.M.A., Toledo A.M., Camargos A.C.R., Longo E., Morais R.L.S., Leite H.R., Palisano R.J., Rosenbaum P. (2024). Functioning profile and related impairments of children and adolescents with cerebral palsy—PartiCipa Brazil preliminary results. BMC Pediatr..

[5] World Health Organization (2003). International Classification of Functioning, Disability and Health.

[6] Elangkovan I.T., Shorey S. (2020). Experiences and needs of parents caring for children with cerebral palsy: A systematic review. J. Dev. Behav. Pediatr..

[7] Smith M., Blamires J. (2022). Mothers’ experience of having a child with cerebral palsy: A systematic review. J. Pediatr. Nurs..

[8] Bistaraki A., Stefanopoulos N., Kiekkas P., Stamatopoulou D., Igoumenidis M. (2025). “I’m not sad anymore, I’m proud to have such a child”: The experiences of caregivers of dependents with cerebral palsy living in Greece. J. Pediatr. Nurs..

[9] Dlamini M.D., Chang Y.J., Nguyen T.T.B. (2023). Caregivers’ experiences of having a child with cerebral palsy: A meta-synthesis. J. Pediatr. Nurs..

[10] Vadivelan K., Sekar P., Sruthi S.S., Gopichandran V. (2020). Burden of caregivers of children with cerebral palsy: An intersectional analysis of gender, poverty, stigma, and public policy. BMC Public Health.

[11] Marques J.S., Regalado I.C., Galvão É.R.V.P., Ferreira H.N., Longo E., Lindquist A.R.R. (2021). Participation in leisure activities from the perception of children with disabilities and their families in Brazil. J. Rehabil. Med..

[12] Steinhardt F., Ullenhag A., Jahnsen R., Dolva A.S. (2021). Perceived facilitators and barriers for participation in leisure activities in children with disabilities: Perspectives of children, parents and professionals. Scand. J. Occup. Ther..

[13] Teleman B., Vinblad E., Svedberg P., Nygren J.M., Larsson I. (2021). Exploring barriers to participation in pediatric rehabilitation: Voices of children and young people with disabilities, parents, and professionals. Int. J. Environ. Res. Public Health.

[14] Minayo M.C.S. (2014). O Desafio do Conhecimento: Pesquisa Qualitativa em Saúde.

[15] Palisano R.J., Rosenbaum P., Bartlett D., Livingston M.H. (2008). Content validity of the expanded and revised Gross Motor Function Classification System. Dev. Med. Child. Neurol..

[16] Associação Brasileira de Empresas e Pesquisa (ABEP) (2024). Critério de Classificação Econômica Brasil (Brazilian Economic Classification Criteria). https://www.abep.org/criterio-brasil.

[17] Eliasson A.C., Krumlinde-Sundholm L., Rösblad B., Beckung E., Arner M., Ohrvall A.M., Rosenbaum P. (2006). The Manual Ability Classification System (MACS) for children with cerebral palsy: Scale development and evidence of validity and reliability. Dev. Med. Child. Neurol..

[18] Eliasson A.C., Ullenhag A., Wahlström U., Krumlinde-Sundholm L. (2017). Mini-MACS: Development of the Manual Ability Classification System for children younger than 4 years of age with signs of cerebral palsy. Dev. Med. Child. Neurol..

[19] Silva D.B., Funayama C.A., Pfeifer L.I. (2015). Manual Ability Classification System (MACS): Reliability between therapists and parents in Brazil. Braz. J. Phys. Ther..

[20] Silva D.B., Pfeifer L.I., Funayama C.A. (2013). Gross Motor Function Classification System Expanded & Revised (GMFCS E & R): Reliability between therapists and parents in Brazil. Braz. J. Phys. Ther..

[21] Bardin L. (2016). Content Analysis.

[22] Alves M.L.F., Souto D.O., Romeros A.C.S.F., Magalhães E.D.D., Mendes L.G., Ayupe K.M.A., Chagas P.S.d.C., de Campos A.C., Moreira R.S., de Toledo A.M. (2024). Characterization of environmental factors in children and adolescents with cerebral palsy in Minas Gerais: Participa Minas. Rev. Paul. Pediatr..

[23] Novak I., Morgan C., Fahey M., Finch-Edmondson M., Galea C., Hines A., Langdon K., Mc Namara M., Paton M.C., Popat H. (2020). State of the Evidence Traffic Lights 2019: Systematic review of interventions for preventing and treating children with cerebral palsy. Curr. Neurol. Neurosci. Rep..

[24] Kalleson R., Jahnsen R., Østensjø S. (2022). Exploring participation in family and recreational activities among children with cerebral palsy during early childhood: How does it relate to motor function and parental empowerment?. Disabil. Rehabil..

[25] Longo E., Regalado I.C.R., Galvão E., Ferreira H.N.C., Badia M., Baz B.O. (2020). I want to play: Children with cerebral palsy talk about their experiences on barriers and facilitators to participation in leisure activities. Pediatr. Phys. Ther..

[26] Mei C., Reilly S., Reddihough D., Mensah F., Green J., Pennington L., Morgan A.T. (2015). Activities and participation of children with cerebral palsy: Parent perspectives. Disabil. Rehabil..

[27] Schiariti V., Sauve K., Klassen A.F., O’Donnell M., Cieza A., Mâsse L.C. (2014). ‘He does not see himself as being different’: The perspectives of children and caregivers on relevant areas of functioning in cerebral palsy. Dev. Med. Child. Neurol..

[28] Fang Z., Cerna-Turoff I., Zhang C., Lu M., Lachman J.M., Barlow J. (2022). Global estimates of violence against children with disabilities: An updated systematic review and meta-analysis. Lancet Child Adolesc. Health.

[29] Syed I.U. (2021). Feminist political economy of health: Current perspectives and future directions. Healthcare.

[30] Armstrong P., Connelly M.P. (1989). Feminist political economy: An introduction. Stud. Political Econ..

