# Supplementary material for: Perceptions of the Body in Cerebral Palsy: Voices of Family Caregivers
Source: Healthcare (Basel). 2026 Apr 7;14(7):967. doi: 10.3390/healthcare14070967 (PMC13072951; doi:10.3390/healthcare14070967)
Supplement: Supplementary file 1 [file healthcare-14-00967-s001.zip › healthcare-4186774-Chart S1.pdf]

SEMISTRUCTURED GUIDE FOR FOCUS GROUPS

Guiding questions (welcoming and familiarization)

1. Tell us a little about your children and grandchildren (if you have more than one) and about your child or grandchild with CP.
2. What do they like to play? Where do they usually play?
3. How was the diagnosis of CP discovered, and how was that experience for you?

Guiding questions (topic guide)

1. What is the daily life of your child or grandchild with CP like?
2. At which moments during activities at home do you support your child or grandchild?
3. Do they go to school? What is their daily routine at school like?
4. What leisure activities does your child or grandchild do outside of home and school? Tell us a little about them.
5. How do you perceive the body of your child or grandchild with CP?
6. Tell us a little about your child's or grandchild's body.
7. Which daily activities at home does your child or grandchild do? How do you participate in these activities?
8. Which daily activities at school does your child or grandchild do? How do you participate in these activities?
9. Which daily activities outside of home does your child or grandchild do? How do you participate in these activities?
10. How do you feel when you do this?
